# Reservoirs of *Staphylococcus* spp. and *Streptococcus* spp. Associated with Intramammary Infections of Dairy Cows

**DOI:** 10.3390/pathogens12050699

**Published:** 2023-05-11

**Authors:** Svenja Woudstra, Nicole Wente, Yanchao Zhang, Stefanie Leimbach, Carsten Kirkeby, Maya Katrin Gussmann, Volker Krömker

**Affiliations:** 1 Department of Veterinary and Animal Sciences, Section for Production, Nutrition and Health, University of Copenhagen, 1870 Frederiksberg, Denmark; 2 Department of Microbiology, Faculty of Mechanical and Bioprocess Engineering, University of Applied Sciences and Arts, 30453 Hannover, Germany; 3 Department of Veterinary and Animal Sciences, Section for Animal Welfare and Disease Control, University of Copenhagen, 1870 Frederiksberg, Denmark

**Keywords:** mastitis prevention, staphylococci, streptococci, pathogen reservoirs, environmental reservoirs, transmission pathways

## Abstract

To design cost-effective prevention strategies against mastitis in dairy cow farms, knowledge about infection pathways of causative pathogens is necessary. Therefore, we investigated the reservoirs of bacterial strains causing intramammary infections in one dairy cow herd. Quarter foremilk samples (*n* = 8056) and milking- and housing-related samples (*n* = 251; from drinking troughs, bedding material, walking areas, cow brushes, fly traps, milking liners, and milker gloves), were collected and examined using culture-based methods. Species were identified with MALDI-TOF MS, and selected *Staphylococcus* and *Streptococcus* spp. typed with randomly amplified polymorphic DNA-PCR. Staphylococci were isolated from all and streptococci from most investigated locations. However, only for *Staphylococcus aureus,* matching strain types (*n* = 2) were isolated from milk and milking-related samples (milking liners and milker gloves). *Staphylococcus epidermidis* and *Staphylococcus haemolyticus* showed a large genetic diversity without any matches of strain types from milk and other samples. *Streptococcus uberis* was the only *Streptococcus* spp. isolated from milk and milking- or housing-related samples. However, no matching strains were found. This study underlines the importance of measures preventing the spread of *Staphylococcus aureus* between quarters during milking.

## 1. Introduction

Mastitis, the inflammation of the mammary gland, is one of the costliest health disorders in dairy farms worldwide [1]. It leads to direct and indirect production losses and can, when flaring up into clinical cases, impair the well-being of affected animals [2,3]. In consequence, constant prevention and control efforts are required in dairy cow herds [3]. 

Mastitis is mostly the result of intramammary infections (IMI) with microorganisms [1]. Staphylococci and streptococci are two bacterial genera comprising several species regularly causing intramammary infections (e.g., *Staphylococcus (Staph.) aureus*, *Staph. epidermidis*, *Streptococcus (Strep). dysgalactiae* and *Strep. uberis*) [4,5,6]. The main habitats of important mastitis pathogens vary due to their distinct requirements towards environmental conditions.

Many staphylococci are skin inhabitants and are therefore often isolated from teat apices and other body parts of dairy cows (e.g., the nares or the hocks), but also from the teat canal [7,8,9]. Consequently, they can be found on milking liners and milkers’ hands or gloves after milking [10]. However, they can also be isolated from the housing environment including locations, such as bedding material, slatted floors, or air samples [11]. For mastitis management, a distinction is usually made between *Staph. aureus* and other staphylococci (non-aureus staphylococci, NAS). *Staph. aureus* is regarded as a major pathogen that causes significant somatic cell count elevations and production losses [12,13]. It is mainly transmitted contagiously from infected to uninfected udder quarters [14]. Therefore, its primary reservoirs are infected quarters and milking-related niches, such as milking liners, milker hands, or gloves [15]. However, *Staph. aureus* can also be found in the respiratory tract of milking personnel or on the teat skin [16]. In contrast to the established relevance of *Staph. aureus* for mammary gland health and milk production, the importance of infections with NAS species remains under constant discussion. At least infections with some species seem to have a significant effect on the somatic cell count in the milk of affected quarters and associations with milk production losses have been found [13,17]. Still, research on the reservoirs of NAS species within dairy herds is limited to a few farms and geographic locations, while considerable differences in species distribution in milk samples between herds and regions have been reported [18]. 

Streptococci are frequently found on mucosal surfaces, but can also regularly be isolated from the skin of animals and humans [19]. The most important species for udder health are *Strep. agalactiae, Strep. dysgalactiae,* and *Strep. uberis*, with the first being nowadays only rarely isolated from the milk of dairy cows in most countries with highly developed dairy production [5,6]. *Strep. uberis* and *Strep. dysgalactiae* have been isolated from different niches in the cows’ environment, including bedding material, drinking troughs, and walking alleys in the barn or pasture [20,21]. 

Knowledge about the reservoirs of mastitis pathogens and their infection pathways is necessary for farmers to improve their prevention management in a cost-effective manner. Preventive measures usually target reduced exposure to mastitis-causing organisms. For pathogens spreading contagiously from quarter to quarter, this can be accomplished with targeted measures like improved milking hygiene or through the separation or culling of infected cows [22,23]. However, preventing IMI due to transmitting pathogens from the environment into subsequently infected quarters remains challenging. Often an improvement of the general hygiene of the animals and the barn is advised to farmers having problems with so-called “environmental pathogens”. However, to use a cost-effective approach, farmers need to know the most important reservoirs harboring mastitis-causing strains. Therefore, increasing our knowledge of potential reservoirs and specifically identifying locations in the environment that harbor those strains that also cause intramammary infections, might help refine preventive measures against new infections with pathogens residing in the cows´ environment. Such studies remain scarce for at least some mastitis-causing organisms (e.g., *Staph. epidermidis*, *Strep. dysgalactiae*, and *Strep. uberis*). 

The objective of this study was therefore to investigate potential reservoirs of selected mastitis-causing staphylococci and streptococci in the housing and milking environment of dairy cows by comparing strain types isolated from milk to those found in samples from different locations in the barn and the milking environment. 

## 2. Materials and Methods

The study population and study design have been previously described in detail [24].

### 2.1. Study Herd

The study farm was located in Sweden and kept approximately 200 lactating cows. Between the 18th of June and the 22nd of October 2020 this farm was visited ten times with 14-day intervals. All lactating cows were milked twice daily in a two times 12 units herringbone milking parlor (GEA Euro class 800 with Dematron 75, GEA Farm Technologies, Bönen, Germany). They were housed in a free stall barn with raised cubicles cushioned with rubber mats and covered with sawdust. The cows had also access to pasture. According to their lactation stage, cows were separated into two groups (group 1: cows until approximately 150 days in milk; group 2: cows in later lactation). During the dry period, cows were either on pasture (separated from lactating cows) or housed in a separate building, depending on the weather conditions. Shortly before calving, cows were moved to a deep straw calving pen. 

During the study period, the herd had a bulk tank somatic cell count of 195,000 cells/mL (geometric mean) and a clinical mastitis incidence of 1.6 cases/100 cows per month among all cows in the herd (lactating and dry) [25]. 

### 2.2. Sample Collection 

#### 2.2.1. Milk Samples

Individual quarter foremilk samples were collected at each of the ten visits from all lactating cows during afternoon milking. In accordance with German Veterinary Association (GVA) guidelines, teat ends were disinfected with disposable paper towels soaked with ethanol (70%), then three milk streams were discarded and finally foremilk was milked into a sterile tube containing Ly-20 [26,27]. 

#### 2.2.2. Milking- and Housing-Related Samples

Milking- and housing-related samples were collected at each of the ten visits as described in Woudstra et al. (2023) [24]. In brief: at every visit, four bedding material samples were collected in sterile 2-liter plastic bags at four locations in the barn of the lactating cows. Each sample contained bedding material from the rear third of four adjacent cubicles [20]. Additionally, at every visit used bedding material from the calving pen was collected in one 2-liter plastic bag. Additionally, samples from the walking alley in front of two drinking troughs and soil from the narrowest point of the passage to the pasture were collected with sterile spoons and transferred into sterile plastic bags. No pasture sample was collected at the last visit, as weather conditions didn´t allow cows access to the pasture anymore.

Using the wet-dry swabbing technique according to DIN10113-1 with slight modifications as described previously, we sampled at each visit the following locations [20,24,28]: the upper surface of drinking troughs, the inner surface of all four milking liners of one milking cluster, as well as the walking alley of the waiting and exit area of the milking parlor. All samples associated with milking (liner and walking alley samples) were collected twice at each visit, once after the first group had completed milking and again after the second group had passed through the milking parlor. At the same time points, also the gloves of the milker were collected and placed in sterile plastic containers. Additionally, two cow brushes were sampled at the approximate height of the cows’ teats using a modified wet-dry swabbing technique [24]. However, these samples were only collected at the last four visits as these brushes had been installed only by then. 

Finally, we also hung up four commercial sticky flytraps at one visit, collected those 14 days later at our next visit, and placed them individually in sterile plastic containers. 

All collected samples were immediately cooled and transported to the laboratory (University of Applied Sciences and Arts Hannover, Germany) and microbiological analysis commenced within 18 h after sampling.

### 2.3. Microbiological Analysis 

#### 2.3.1. Milk Samples 

Microbiological analysis of milk samples was conducted according to the GVA guidelines and as described previously [25,27]. Of each sample, 10 µL were streaked onto esculin blood agar plates (Oxoid Inc., Wesel, Germany). These were then incubated aerobically at 37 °C and bacterial growth was examined after 24 and 48 h. Milk samples with growth of more than two distinct colony types were considered contaminated. Preliminary species identification was carried out based on morphological and biochemical characteristics. For final species identification with MALDI-TOF MS individual colonies were picked and incubated for 24 h at 37 °C on esculin blood agar.

#### 2.3.2. Milking- and Housing-Related Samples

The microbiological analysis of milking- and housing-related samples has been described previously in detail [24]. An overview of the applied sample processing, the used culture media per sample type and the respective incubation periods can be found in Table 1. Of each sample, up to 24 differently looking colonies were transferred onto new esculin blood agar plates after incubation to produce pure cultures. These esculin blood agar plates were then incubated aerobically for 24 h at 37 °C. 

### 2.4. Species Identification with MALDI-TOF MS

From all pure cultures sub-cultivated from all sample types, colony material was directly smeared onto a MALDI-TOF MS steel target according to the manufacturer’s instructions (Bruker Daltonik, Bremen, Germany). All isolates were analyzed by mass spectrometry (Microflex LT/SH smart, Bruker Daltonik, Bremen, Germany) and resulting spectra compared to the MBT Compass Library (Version 9, Revision F, MBT 8468 MSP Library). MALDI scores of ≥1.7 and ≥2.0 were considered a secure genus and species identification, respectively [29]. Isolates with a score below 1.7 were considered to be “not identified”. Finally, from all pure cultures one colony was picked and stored in a solution containing 80% brain heart infusion and 20% glycerol at −80 °C.

### 2.5. RAPD PCR (Randomly Amplified Polymorphic DNA PCR)

All *Staph. aureus*, *Staph. epidermidis*, *Staph. haemolyticus*, *Strep. uberis,* and *Strep. dysgalactiae* strains isolated from any sample type were eligible for typing by RAPD-PCR. The species selection was based on the frequency of isolation from milk samples and the average somatic cell count of milk samples positive for the respective species (Table 2 and Table 3, somatic cell count data was reported in [25]).

From all stored pure cultures of the above-mentioned species, DNA was isolated using the DNeasy Blood & Tissue Kit (Qiagen, Venlo, The Netherlands). The PCR protocol and primers reported by Woudstra et al. (2023) were used for the RAPD-PCR [25]. In brief, primer ERIC 1R was used for *Staph. epidermidis* isolates, primer C was used for *Staph. aureus* and *Staph. haemolyticus*, and primer OPE 04 was used for *Strep. dysgalactiae* and *Strep. uberis* [30,31,32]. The PCR reaction mix contained 12.5 μL ReadyMix™ Taq PCR Reaction Mix (SigmaAldrich, Munich, Germany), 20 pmol of the respective primer, and water to fill up to a volume of 20 µL per reaction tube. Five µL of template DNA was added to the PCR reaction mix and amplifications run in an Mx3005 P qPCR System (Agilent, Santa Clara, CA, USA). MIDORIGreen Direct (NIPPON Genetics Europe GmbH, Düren, Germany) was added to the PCR products, and separation was carried out on 2% agarose gels. Subsequently, gel pictures were taken with the InGenius LHR system, and the banding pattern was analyzed with GeneTools (both Syngene, Cambridge, UK). Isolates of the same species were considered to belong to the same RAPD type if banding patterns were identical [33]. To confirm that isolates belonged to the same RAPD type, those with identical banding patterns were analyzed a second time by running their PCR products next to each other on new 2% agarose gels.

## 3. Results

In total, 8056 milk samples and 251 milking- or housing-related samples were collected (Table 2).

### 3.1. Microbiological Results

The *Staphylococcus* spp. most frequently isolated from milk samples were in declining order *Staph. chromogenes*, *Staph. epidermidis*, *Staph. aureus*, *Staph. haemolyticus* and *Staph. simulans* (Table 2), while *Staph. capitis*, *Staph. cohnii*, *Staph. gallinarum*, *Staph. hominis*, *Staph. hyicus*, *Staph. saprophyticus*, *Staph. sciuri*, *Staph. succinus,* and *Staph. xylosus* were only rarely isolated from milk. All *Staphylococcus* spp. were isolated from less than 2% of all milk samples throughout the sampling period (Table 2). The 86 *Staph. aureus* isolates originated from 45 quarters of 38 cows [25]. Furthermore, the 96 *Staph. epidermidis* and 71 *Staph. haemolyticus* isolates originated from 33 and 49 quarters of 26 and 40 cows, respectively. 

*Staphylococcus* spp. were isolated from ≥90% of all samples from milking liners (76/80), milker gloves (18/20), drinking troughs (19/20), bedding in the close-up pen (9/10), fly traps (4/4) and cow brushes (8/8) (Table 2). Furthermore, ≥50% of all samples from bedding of lactating cows (29/40) and the floor in the waiting area of the milking parlor (10/20) were positive for staphylococci, while they were isolated less often from the exit of the milking parlor (4/20) and the access to pasture (1/9). *Staph. aureus* was only isolated from samples associated with milking (milking liners (*n* = 7) and milker gloves (*n* = 2)). On the other hand, 8 of the 14 NAS species isolated from either milking- or housing-related samples were found in both the housing and milking environment of the cows. Only *Staph. chonii* (*n* = 3), *Staph. petrasii* (*n* = 1), *Staph. saprophyticus* (*n* = 2), and *Staph. simulans* (*n* = 1) were not isolated from samples directly associated with milking (i.e., milking liners or milker gloves). The only *Staphylococcus* species that were solely isolated from milk samples were *Staph. gallinarum* (*n* = 1), *Staph. hyicus* (*n* = 6), and *Staph. succinus* (*n* = 1). 

Streptococci were isolated from fewer housing-related samples than staphylococci and only one time from samples directly associated with milking (one milking liner, Table 3). They were mainly isolated from bedding material in the lactating cow pen (*n* = 6) and associated with the drinking trough (*n* = 5). *Strep. uberis* was frequently isolated from milk samples (*n* = 115 of 29 quarters from 21 cows, [25]) and found in one bedding material and one milking liner sample. The other three streptococci isolated from milk (*Strep. dysgalactiae (n* = 107 of 33 quarters from 28 cows, [25]), *Strep. canis,* and *Strep. gallolyticus*) were not isolated from any sample collected from milking- or housing-related niches. All *Streptococcus* spp. were isolated from less than 2% of all milk samples throughout the sampling period (Table 3).

### 3.2. Strain Typing Results

As *Strep. dysgalactiae* could not be isolated from any of the milking- or housing-related samples, no comparison of strain types from milk and environment could be carried out for this species. For *Staph. aureus*, *Staph. epidermidis*, *Staph. haemolyticus,* and *Strep. uberis* all isolates cultivated from the milking- or housing-related samples or milk were typed by RAPD PCR. The complete strain typing results of milk samples have been reported previously [25]. Here, the focus lies on strain types isolated from milking- and housing-related samples and their comparison to strains isolated from milk.

For *Staph. aureus,* two different strain types were isolated from milker gloves and milking liners (Table 4). Both strain types were also found in milk samples on the same date they were isolated from milking-related samples (Table 4, date of isolation not shown). The dominant strain in milk samples (type A) was also the one most frequently isolated from milking-related niches. Still, strain type D could be cultivated from a milking liner, although on that day only one quarter tested positive for the same strain. One *Staph. aureus* isolated from a milking liner sample could not be recovered after storage in glycerol and was therefore not strain typed. 

In contrast, nine different *Staph. epidermidis* strains were isolated from milking- and housing-related samples including samples from bedding material, milking liners, milker gloves, and fly traps (flies and mosquitos), but none of the isolated strains were also found in milk samples. For Staph. *haemolyticus,* an even higher strain diversity with 55 different strains was found in milking- and housing-related samples. *Staph. haemolyticus* strains were isolated from milker gloves, milking liners, bedding material, cow brushes, and drinking troughs. Again, none of the strains matched with one of the 69 strains isolated from milk (Table 4).

From each of one milking liner sample and one bedding material sample, one strain of *Strep. uberis* was isolated (Table 4). Both strains were not isolated from milk samples.

## 4. Discussion

The present study describes species-level reservoirs of staphylococci and streptococci in the housing and milking environments of dairy cows. However, the primary aim was to identify in more depth the reservoirs of those strains of selected species (*Staph. aureus*, *Staph. epidermidis*, *Staph. haemolyticus*, *Strep. dysgalactiae,* and *Strep. uberis*) that caused intramammary infections during the study period. 

### 4.1. Staphylococci

*Staphylococcus* spp. were isolated at least once from all investigated niches and from many locations several different species were isolated over the study period. Especially surfaces that are often in contact with the skin of animals or their feces (e.g., bedding material, the drinking trough, milking liners, and the gloves of the milker) were potential reservoirs of staphylococci. 

Concerning milking- and housing-related samples, *Staphylococcus aureus* was only isolated from milking-related niches (milker gloves and milking liners). Two different strains were isolated from these locations and both strains were also cultivated from at least one milk sample on the respective sampling day. One of these two strains was clearly dominant in both sample types, milking-related and milk samples. The presence of a dominant strain in milk samples indicates that a species is mainly transmitted contagiously from quarter to quarter [34]. Similar indications of mainly contagious transmission of *Staph. aureus* have been previously reported in several studies [14,16,35]. That *Staph. aureus* was furthermore only isolated from samples associated with milking indicates additionally that the main reservoirs of this species were infected udder quarters. However, it was proposed that the finding of numerous strains of *Staph. aureus* in dairy herds, of which some are found only rarely, indicates that *Staph. aureus* spreads not only contagiously from cow to cow, but also via environmental routes [34]. In contrast, our results indicate that even strains with a low prevalence (here type D) can be isolated from milking-related niches and might still spread during milking from quarter to quarter. 

Both *Staph. epidermidis* and *Staph. haemolyticus* had a very high diversity in environmental (milking- and housing-related) and milk samples in the current study (Table 4, [25]), and none of their milking- or housing-related isolates matched with any of the isolates cultivated from milk samples. These findings underline the environmental nature of infections with these two NAS species in the study herd. However, they also demonstrate how difficult it can be to identify the actual reservoirs of those strains causing mastitis via environmental routes. 

*Staph. epidermidis* is frequently isolated from human skin, and it was proposed that human skin might be the major source of intramammary *Staph. epidermidis* infections [36]. In this study, *Staph. epidermidis* was isolated from various sources including the inner surface of the milking liner, the outside of the milkers´ gloves, and bedding material. All of these likely had more contact with cow skin than human skin. Furthermore, Wuytack et al. demonstrated that *Staph. epidermidis* can be regularly isolated from teat apices of dairy cows and rectal fecal samples [8]. In the same study, *Staph. epidermidis* was never isolated from teat apices before milking, but several times after milking. This indicates that *Staph. epidermidis* might be flushed onto the teat skin and milking liners during milking. In contrast, for other NAS species, it has been suggested that they are washed away during milking or are strongly reduced through the application of a pre-milking disinfection [8]. Although we found *Staph. epidermidis* on milking liners and milker gloves after milking, we never isolated the same strain type from both milk and milking-related samples, suggesting that transmission may be sporadic. In contrast, Piessens et al. were able in one out of six herds to isolate the same strain from milk as from alleyways and bedding material [37]. The authors of that study proposed that the housing environment might not be an important reservoir for *Staph. epidermidis*. Additionally, the findings of our study and those of Wuytack et al. (2020) suggest that mainly surfaces with regular skin contact might belong to the important fomites for *Staph. epidermidis* transmission. 

We isolated *Staph. epidermidis* also from fly traps (Table 2). Flies and mosquitos could therefore be involved in the transmission of this microorganism. For *Staph. aureus,* it has been proven experimentally that flies can be a source of new intramammary infections [38]. Additionally, in an observational study, the same genotypes were found in cows’ milk, heifers’ colostrum, and horn flies [39]. Although we did not detect matching strain types of *Staph. epidermidis* in milk and fly samples, we hypothesize that flies could also be mechanical vectors for *Staph. epidermidis* subsequently leading to intramammary infections. Further research could elucidate the role of flying insects in the transmission of *Staph. epidermidis*. 

*Staph. haemolyticus* was isolated in the present study from bedding material from the lactating cow pen, the surface of drinking troughs, cow brushes, milking liners, and milker gloves. This species has also previously been isolated from housing-related niches like alleyways, barn air, bedding material, and sawdust in storage, as well as from cow-related niches, such as the teat apex or rectal feces [8,37]. In the present study, most of the investigated *Staph. haemolyticus* isolates originated from milking liner samples. These are in close contact to the teats during milking. Wuytack et al. (2020) isolated *Staph. haemolyticus* from up to 100% of all sampled teat apices before milking, while after milking the proportion of positive teat apices decreased [8]. The same authors also found the same RAPD type in quarter milk and teat apex samples [33]. Interestingly, the teat apex samples and milk samples harboring the same strain never came from the same quarter or cow. 

Furthermore, Piessens et al. found in each of the six herds at least one strain of *Staph. haemolyticus* that could be isolated from both, milk and environmental samples (e.g., from alleyways, air, or bedding material) [37]. In the present study, we were able to detect two *Staph. haemolyticus* RAPD types twice in milking-related samples collected at different sampling dates. However, we found no match between these isolates (*n* = 57) and those obtained from milk (*n* = 71). One difference between our study and Piessens et al. (2021) is that our milking- or housing-related *Staph. haemolyticus* isolates mainly originated from milking liners and milker gloves, while Piessens et al. (2021) mainly recovered the matching isolates from alleyway samples. Furthermore, we used the same definition to distinguish RAPD types as Piessens et al. (2021) and Wuytack et al. (2020), but a different RAPD primer (primer C) than the two mentioned studies (primer D11344, [33,37]). The primer used in the present study may have been more discriminative for *Staph. haemolyticus* than the primer used in the two previous investigations. However, we did not test our samples with primer D11344 which would have enabled us to compare RAPD profiles resulting from using different primers. Yet, Piessens et al. have applied a combination of AFLP and RAPD typing to determine strain types which should have improved the discriminatory power compared to using each typing method alone [37].

### 4.2. Streptococci

*Streptococcus* spp. were isolated in our study from used bedding material of lactating cows, drinking troughs, and the floor in front of these, one cow brush, the passage to pasture, the waiting area, and one milking liner. In comparison to staphylococci, we isolated streptococci less frequently. 

Of those *Streptococcus* species isolated from milk samples, we only cultivated *Strep. uberis* also from milking- or housing-related samples (once from bedding material and once from a milking liner). Previously, *Strep. uberis* has been isolated from many different areas in dairy farm environments, for example from drinking troughs, bedding material, and outdoor laying areas, the passage to pasture, the waiting and exit area of the milking parlor as well as milking liners [20,40,41,42]. While in the present study, none of the milking- or housing-related isolates belonged to a strain type also found in milk samples, a previous study investigating potential sources of *Strep. uberis* isolates from clinical mastitis cases found the same strains in milk as on a drinking trough, the waiting area of the milking parlor, the passage to pasture, and a milking liner [20]. The respective study investigated 103 environmental strains from 15 different farms of which four matched with isolates from clinical mastitis cases. Therefore, it is not surprising that with only two *Strep. uberis* isolates recovered in this study, it was unlikely to find a match with strains from milk. The samples of the present study were analyzed in the same laboratory using a similar approach as described by Wente et al. (2019). However, the previous study specifically searched only for *Strep. uberis* in milking- and housing-related niches, while in this study in total up to 24 differently looking colony types were picked from each sample. 

The strain typing of *Strep. uberis* isolated from milk samples from the study herd indicated that *Strep. uberis* had likely been transmitted partly contagiously between udder quarters as each 24%, 14%, and 11% of all *Strep. uberis* infections had been caused by one distinct strain type only [25]. However, the predominance of strains in infected quarters can also indicate the presence of environmental hotspots. Unfortunately, the present study could not shed further light on the origin (contagious transmission or environmental hotspot) of *Strep. uberis* infections that were linked through the same strain type within the study herd. 

We did not detect *Strep. dysgalactiae* in any of the milking- and housing-related samples while we isolated it from 107 milk samples throughout the study. One reason could be that we did not sample the skin of teats and udder, or skin wounds that were found in a study (published after the completion of the present study) to be major sources of *Strep. dysgalactiae* [21]. In that study, *Strep. dysgalactiae* could not be cultured, e.g., from any of the fecal samples. The authors used a two-step procedure for the isolation of *Strep. dysgalactiae* from environmental samples: first, a qPCR was conducted, and only qPCR-positive samples underwent microbiological examination on a selective medium with culture under anaerobic conditions. In this study, we used less selective methods, because the intention of the overall study was to investigate the infection dynamics and transmission routes of several mastitis pathogens within the same herd. While our approach was sensitive enough for recovering many *Strep. dysgalactiae* isolates from milk samples, the competitive flora in milking- and housing-related samples might have been too strong for *Strep. dysgalactiae* to grow. 

### 4.3. Study Design 

One limitation of the present study is that it was carried out in one dairy cow herd only. However, we collected samples at 10-time points over a period of 18 weeks and not only investigated reservoirs on species but also on strain level. For some of the studied pathogens, only very limited data exists on strain comparisons for isolates recovered from milking- and housing-related niches to those cultivated from milk samples (especially *Staph. epidermidis* and *Strep. dysgalactiae*). Therefore, the present study adds to the existing literature and provides further insights into the potential transmission routes of several mastitis pathogens. One could also argue that we could have sampled additional sites (e.g., the skin of animal caretakers or further locations in the barn). However, we had to select sites to investigate due to financial limitations and based our choice on previously published literature [10,11,20,39]. In addition, the microbiological procedures were carefully chosen and included the inoculation of samples on selective media (i.e., Baird Parker Agar for *Staphylococcus* spp. and Edwards modified medium for *Streptococcus* spp.). However, limiting the number of totally investigated isolates per sample to a maximum of 24 might have led to an underreporting of species found and might have also limited the chance of finding matching strains for species with a very high diversity (e.g., *Staph. haemolyticus*). In addition, a method with a higher sensitivity for the individual studied species (e.g., *Strep. uberis* or *Strep. dysgalactiae*) could have probably led to the recovery of a larger number of strains from milking- and housing-related samples as for example Zadoks et al. 2005 or Smistad et al., 2022 were able to recover *Strep. uberis* or *Strep. dysgalactiae* frequently from various sources [21,40]. 

## 5. Conclusions

Overall, staphylococci, and to a lower extent streptococci, were isolated from many different locations in the housing and milking environment of cows. All identified habitats could be potential reservoirs leading to contamination of teats and consecutive infections. However, for *Staph. epidermidis*, *Staph. haemolyticus* and *Strep. uberis* none of the RAPD profiles of isolates cultivated from milking- or housing-related niches matched with those of isolates from milk. *Staph. aureus* however was only isolated from locations directly related to milking and only isolates with RAPD profiles matching with those from strains isolated from milk were found. This indicates that the main reservoirs of *Staph. aureus* were infected udder quarters and underlines the importance of measures preventing the spread of *Staph. aureus* infections between quarters during milking. 

## Figures and Tables

**Table 1:** Overview of processing and used culture media for milking- and housing-related samples as previously described [24].

Sample Location/Type	Sample Processing	Inoculated Decimal Dilutions	Culture Media (Incubation Time in Hours) ^3^
Bedding material and ground samples (pasture, slatted floors)	sample material (10 g) was transferred into a sterile Stomacher^®^ bag, diluted with 90 mL Ringer’s solution ^1^ and mixed for 1 min	10^−2^–10^−5^	Esculin blood agar ^1^ (48)
			Baird Paker agar ^1^(48)
			Chromocult^®^ Coliform agar ^1^ (24)
			Edwards modified medium agar ^2^ (24)
Tubes with swabs	mixed for 1 min	10^−1^–10^−4^	Esculin blood agar ^1^ (48)
Gloves	fixed with a bag clip at the wrist part to the upper end of a Stomacher^®^ bag containing 100 mL half concentrated Ringer’s solution ^1^ and mixed for 1 min	10^−2^–10^−3^	Baird Paker agar ^1^ (48)
			Chromocult^®^ Coliform agar ^1^ (24)
			Edwards modified medium agar ^2^ (24)
Flytraps	flies belonging to the same species were placed in 2 mL reaction tubes with 1 mL sterile Ringer’s solution ^1^; (a) recovery of microbes from outer surface: tubes were mixed for 10 s and the complete liquid was transferred to a new sterile tube; (b) recovery of microbes inside the flies: subsequently new Ringer’s solution (1 mL) was filled into each tube containing flies, after homogenization, the complete solution was used	10^−3^–10^−5^	Baird Paker agar ^1^ (48)
			Chromocult^®^ Coliform agar ^1^ (24)
			Edwards modified medium agar ^2^ (24)

^1^ Merck, Darmstadt, Germany; ^2^ Oxoid, Wesel, Germany, supplemented with colistin sulfate and oxolinic acid both from Sigma-Aldrich, Munich, Germany; ^3^ all media were incubated at 37 ± 1 °C.

**Table 2:** Isolated *Staphylococcus* spp. from milking- and housing-related samples (*n* = 251) and milk (*n* = 8056) and number of positive samples by sample type.

	Sample Location/Type (*n* * =)											
Species	Bedding Lactating Cows (40)	Bedding Close Up Pen (10)	Drinking Trough (20)	Floor Drinking Trough (20)	Cow Brush (8)	Fly Trap (4)	Passage to Pasture (9)	Waiting Area (20)	Milking Exit (20)	Milking Liner (80)	Milker Gloves (20)	Milk Samples (8056) **
*Staph. aureus*	-	-	-	-	-	-	-	-	-	7	2	86
*Staph. capitis*	1	-	-	-	-	-	-	-	-	-	1	6
*Staph. chromogenes*	-	-	1	-	1	-	-	-	-	1	5	133
*Staph. cohnii*	1	-	-	-	1	1	-	-	-	-	-	1
*Staph. epidermidis*	1	-	-	-	-	2 ***	-	-	-	1	1	96
*Staph. equorum*	-	-	-	-	-	3	-	-	-	2	-	-
*Staph. gallinarum*	-	-	-	-	-	-	-	-	-	-	-	1
*Staph. haemolyticus*	1	-	5	-	3	-	-	-	-	18	8	71
*Staph. hominis*	-	-	2	-	-	1	-	-	-	4	-	1
*Staph. hyicus*	-	-	-	-	-	-	-	-	-	-	-	6
*Staph. pasteuri*	-	-	-	-	-	-	-	-	-	-	3	-
*Staph. petrasii*	-	-	-	-	-	1	-	-	-	-	-	-
*Staph. saprophyticus*	-	-	1	-	-	1	-	-	-	-	-	2
*Staph. sciuri*	6	-	2	3	-	2	-	-	-	7	-	3
*Staph. simulans*	-	-	-	-	-	-	-	1	-	-	-	26
*Staph. succinus*	-	-	-	-	-	-	-	-	-	-	-	1
*Staph. warneri*	-	-	-	-	-	-	-	-	-	-	2	-
*Staph. xylosus*	-	3	-	-	-	3	-	-	-	-	1	1
*Staph.* spp.	26	9	19	8	8	4	1	10	4	74	17	341
Total ****	29	9	19	9	8	4	1	10	4	76	18	773

* total number of samples taken throughout the project from the specific location/sample type; ** milk sample results have been reported previously [25] *** from one fly trap *Staph. epidermidis* was isolated from attached flies and mosquitos and from a second fly trap only from flies; **** total number of samples positive for at least one *Staphylococcus* species.

**Table 3:** Isolated *Streptococcus* spp. from milking- and housing-related samples (*n* = 251) and milk (*n* = 8056) and number of positive samples by sample type.

	Sample Location/Type (*n* * =)											
Species	Bedding Lactating Cows (40)	Bedding Close Up Pen (10)	Drinking Trough (20)	Floor Drinking Trough (20)	Cow Brush (8)	Fly Trap (4)	Passage to Pasture (9)	Waiting Area (20)	Milking Exit (20)	Milking Liner (80)	Milker Gloves (20)	Milk Samples (8056) **
*Strep. dysgalactiae*	-	-	-	-	-	-	-	-	-	-	-	107
*Strep. canis*	-	-	-	-	-	-	-	-	-	-	-	4
*Strep. gallolyticus*	-	-	-	-	-	-	-	-	-	-	-	2
*Strep. lutetiensis*	-	-	-	1	-	-	-	-	-	-	-	-
*Strep. parauberis*	-	-	-	-	-	-	-	1	-	-	-	-
*Strep. uberis*	1	-	-	-	-	-	-	-	-	1	-	115
*Strep.* spp.	5	-	2	2	1	-	1	1	-	-	-	35
Total ***	6	-	2	3	1	-	1	2	-	1	-	262

* total number of samples taken throughout the project from the specific location/sample type; ** milk sample results have been reported previously [25]; *** total number of samples positive for at least one *Streptococcus* species.

**Table 4:** *Staphylococcus* and *Streptococcus* spp. strains isolated from milking- and housing-related samples.

Species	Strains Isolated from Milking- and Housing-Related Samples *	Sample Location (*n* Positive Samples from Location)	No of Milk Samples Positive for Respective Strain
Staph. aureus **	A	Milker gloves (2), Milking liner (5)	67
	D	Milking liner (1)	5
*Staph. epidermidis*	EA	Milking liner (1)	0
	EB	Milking liner (1)	0
	EC	Milking liner (1)	0
	ED	Milker gloves (1)	0
	EE	Milker gloves (1)	0
	EF	Bedding lactation pen (1)	0
	EG	Fly trap (1) ^5^	0
	EH	Fly trap (1) ^5^	0
	EI	Fly trap (1) ^6^	0
*Staph. haemolyticus*	EA	Milking liner (2)	0
	EB	Milking liner (2)	0
	EC–ES ^1^	Milking liner (each 1)	0
	ET–EAM ^2^	Milker gloves (each 1)	0
	EAN–EAT ^3^	Cow brush (each 1)	0
	EAU–EBB ^4^	Drinking trough (each 1)	0
	EBC	Bedding lactation pen (1)	0
*Strep. dysgalactiae*	none	-	-
*Strep. uberis*	S	Bedding lactation pen (1)	0
	T	Milking liner (1)	0

* Strain names were continuously assigned throughout the overall project [25]. Due to the large diversity among *Staph. epidermidis* and *Staph. haemolyticus* strains names of milking and housing-related strains start with “E” + letters in alphabetical order; ** one isolate of *Staph. aureus* could not be recovered from the frozen glycerol tube, ^1^ including strains: EC, ED, EE, EF, EG, EH, EI, EJ, EK, EL, EM, EN, EO, EP, EQ, ER, and ES; ^2^ including strains: ET, EU, EV, EW, EX, EY, EZ, EAA, EAB, EAC, EAD, EAE, EAF, EAG, EAH, EAI, EAJ, EAK, EAL, and EAM; ^3^ including strains: EAN, EAO, EAP, EAQ, EAR, EAS, EAT; ^4^ including strains: EAU, EAV, EAW, EAX, EAY, EAZ, EBA, EBB; ^5^ flies; and ^6^ mosquitoes.

## Data Availability

Not applicable.
